# MST1/2 exerts a pivotal role in inducing neuroinflammation and Coxsackievirus-A10 replication by interacting with innate immunity

**DOI:** 10.1186/s12985-024-02355-5

**Published:** 2024-04-19

**Authors:** Yajie Hu, Minigmei Zhong, Yaming Lv, Wei Zhao, Baojiang Qian, Jie Song, Yunhui Zhang

**Affiliations:** 1 Department of Respiratory Medicine, The First People’s Hospital of Yunnan Province, Kunming, China; 2 The Affiliated Hospital of Kunming University of Science and Technology, Kunming, China; 3 Institute of Medical Biology, Chinese Academy of Medical Science and Peking Union Medical College, Kunming, China

**Keywords:** Hand, foot and mouth disease (HFMD), Coxsackievirus-A10 (CV-A10), Neuroinflammation, Hippo signaling pathway, Mammalian Ste20-like kinases 1/2 (MST1/2)

## Abstract

Coxsackievirus-A10 (CV-A10), responsible for the hand, foot and mouth disease (HFMD) pandemic, could cause serious central nervous system (CNS) complications. The underlying molecular basis of CV-A10 and host interactions inducing neuropathogenesis is still unclear. The Hippo signaling pathway, historically known for a dominator of organ development and homeostasis, has recently been implicated as an immune regulator. However, its role in host defense against CV-A10 has not been investigated. Herein, it was found that CV-A10 proliferated in HMC3 cells and promoted the release of inflammatory cytokines. Moreover, pattern recognition receptors (PRRs)-mediated pathways, including TLR3-TRIF-TRAF3-TBK1-NF-κB axis, RIG-I/MDA5-MAVS-TRAF3-TBK1-NF-κB axis and TLR7-MyD88-IRAK1/IRAK4-TRAF6-TAK1-NF-κB axis, were examined to be elevated under CV-A10 infection. Meanwhile, it was further uncovered that Hippo signaling pathway was inhibited in HMC3 cells with CV-A10 infection. Previous studies have been reported that there exist complex relations between innate immune and Hippo signaling pathway. Then, plasmids of knockdown and overexpression of MST1/2 were transfected into HMC3 cells. Our results showed that MST1/2 suppressed the levels of inflammatory cytokines via interacting with TBK1 and IRAK1, and also enhanced virus production via restricting IRF3 and IFN-β expressions. Overall, these data obviously pointed out that CV-A10 accelerated the formation of neuroinflammation by the effect of the Hippo pathway on the PRRs-mediated pathway, which delineates a negative immunoregulatory role for MST1/2 in CV-A10 infection and the potential for this pathway to be pharmacologically targeted to treat CV-A10.

**Supplementary Information:**

The online version contains supplementary material available at 10.1186/s12985-024-02355-5.

## Introduction

Hand, foot and mouth disease (HFMD) is generally a benign febrile exanthematous childhood illness that most typically noted in children younger than 10 years old, primarily caused by human enteroviruses, mainly including enterovirus 71 (EV-A71), coxsackievirus A16 (CV-A16), coxsackievirus-A10 (CV-A10) and coxsackievirus-A6 (CV-A6) [1]. The illness usually begins with a prodrome of fever, malaise, poor appetite, and sore throat, then exhibits skin eruptions on palms, fingers, toes, soles, buttocks, and ulcers or blisters in the mouth, with symptoms usually lasting 7∼10 days [1, 2]. However, a small proportion of children may experience severe neurological syndromes, which can further progress to cardiopulmonary complications and even death [2, 3]. Now, three inactivated monovalent EV-A71 vaccines were developed and licensed in China. Each of them has shown a high level of safety and immunogenicity against EV-A71-associated HFMD, but none offers cross-neutralizing protection against other HFMD-causing viral strains [4, 5]. Thus, developing a multivalent inactivated vaccine which can target major prevalent pathogenic enteroviruses remain the most effective way to prevent and control HFMD. Based on current epidemiological data, the proportion of sporadic HFMD cases and outbreak events caused by CV-A10 has been increasing worldwide in recent years [6, 7]. CV-A10, a human enterovirus belonging to the A species of the Enterovirus genus within Picornaviridae family, has been found to be a serious underestimate of its harm [8]. CV-A10 infection usually cause more severe forms of HFMD [9]. Moreover, due to the variability of the CV-A10 genome, the co-infection of CV-A10 with other multiple enteroviruses results in an increasing of recombination events, which has aroused widespread concern and attention of the research community worldwide [8]. Hence, the study of the pathogenesis of CV-A10 can not only enrich the theoretical mechanism of CV-A10 infection, but also provide a new theory for the development of multivalent vaccines.

Hippo signaling pathway is an evolutionarily conserved signaling cascade modulating the proliferation, differentiation and survival of cells [10]. As such, this pathway is closely associated with the control of organ size, cancer development, and tissue regeneration. The canonical Hippo signaling pathway is composed of more than 30 components, but there are three core components: mammalian Ste20-like kinases 1/2 (MST1/2), large tumor suppressor 1/2 (LATS1/2) and Yes-associated protein (YAP) or a transcriptional coactivator with PDZ-binding motif (TAZ). In recent years, emerging discoveries elucidated that the core components of Hippo signaling pathway were involved in the immunity regulation [10, 11]. For example, MST1 suppressed the activation of TANK-binding kinase 1 (TBK1), which avoid excessive activation of the downstream innate antiviral immunity [12]. MST1 also mediated the degradation of IL-1R-associated kinase 1 (IRAK1), which further inhibited TLR4/9-NF-κB signaling and inflammatory responses [13]. YAP/TAZ was documented to inhibit host anti-viral immunity by blocking the function of TBK1 [13, 14]. Meanwhile, MST1/2 regulated the adhesion and trafficking of antigen-presenting cells and also involved in in the development of dendritic cells, B cells, and T cells [15]. Furthermore, it was reported that MST1/2 enhanced Treg differentiation via promoting Foxp3’s acetylation and activity, while TAZ had been found to facilitate helper T cell differentiation but impede Treg cell differentiation [16, 17]. Thus, there were a close crosstalk between Hippo signaling pathway and innate immunity or adaptive immunity.

Current findings revealed the key function of the Hippo signaling pathway in virus infection and pathogenesis [18, 19]. For instance, severe acute respiratory syndrome coronavirus type 2 (SARS-CoV-2) infection activated TLR4 leading to the release of pro-inflammatory cytokines, which in turn initiated Hippo signaling pathway to exaggerate the cytokine storm [20]. The nonstructural protein 4B (NS4B) of hepatitis C virus (HCV) induced lipogenesis via mediating Hippo signaling pathway, which finally resulted in hepatic steatosis and even hepatocellular carcinoma [21]. Zika virus (ZIKV) infection might trigger a cross talk between AMP-activated protein kinase and Hippo-TBK1 pathways, which could exert a critical role in viral replication and the process of neuroinflammation [22]. Thence, it was guessed whether there might be a link between CV-A10 infection and Hippo signaling pathway.

## Materials and methods

### Cell culture, virus infection and transfection

Human microglia cells (HMC3; Procell, China) and Vero cells were cultivated in Dulbecco’s Modified Eagles Medium (DMEM) containing 10% fetal bovine serum (FBS) and 100 U/ml penicillin-streptomycin at 37 °C with 5% CO_2_.

The CV-A10 (subgenotype C, GenBank NO. MN557275), which was isolated during an epidemic in Xiangyang, China, in 2017, were proliferated in Vero cells for this investigation. To explore the effect of CV-A10 on glial cells, HMC3 cells were plated into 6-well plates. Next day, CV-A10 was inoculated into cells at a multiplicity of infection (MOI) of 0.1 for 2 h at 37 °C. Subsequently, the virus inoculum was discard and the maintenance medium (i.e., DMEM containing 2% FBS) was replaced. Cells and supernatants were collected at the indicated time points.

In order to further investigate the biological role of MST1/2, the overexpression and knockdown vectors of MST1/2, as well as the corresponding negative control, were constructed, synthesized and purchased from Genepharma (Shanghai, China). These vectors were transfected into HMC3 cells using Lipofectamine 3000 (Invitrogen, USA) according to the standard procedure. Following a 24 h transfection, the cells were used for subsequent experiments.

### Quantitative real-time polymerase chain reaction(qRT-PCR)

The quantitation of the number of copies of the CV-A10 genome was achieved by qRT-PCR. Briefly, total RNA was extracted using TIANamp Virus RNA Kit (TIANGEN, China) according to the manufacturer’s instructions. Subsequently, a commercial Coxsackievirus A10 RNA detection kit (TIANLONG, China) was used to detect the viral load with a GENTIER 96 equipment (TIANLONG, China). Meanwhile, the standards for CV-A10 with known concentrations were concurrently measured with the test samples. Finally, the viral load of CV-A10 in each sample was calculated according to the standard curve.

### Titration of viruses

Viral titers were determined by 50% tissue culture infectious dose (TCID_50_) analysis on Vero cells using the Reed-Muench method. The supernatants of the infected culture were collected at indicated time and serially diluted in serum-free DMEM. Ten-fold diluted culture was adsorbed onto confluent Vero cells in a 96-well plate for 2 h at 37 °C. Later, the inoculum was removed, infected cells were washed with sterile phosphate-buffered saline (PBS) and cultured with in DMEM with 2% FBS for 3∼5 days.

### Western blotting (WB)

Cells infected with CV-A10 for the indicated time were lysed in RIPA lysis buffer (Beyotime, China) with protease/phosphatase inhibitor Cocktail (Boster, China) on ice for 30 min. A BCA protein quantification kit (Beyotime, China) was used to determine the concentration of extracted proteins. 30 micrograms of proteins were resolved via 12% sodium dodecyl sulfate polyacrylamide gel electrophoresis (SDS-PAGE) and transferred to the polyvinylidene difluoride (PVDF) membrane. 5% nonfat milk was used for transferred PVDF membranes block for 1 h at room temperature, and then the membranes were incubated overnight at 4 °C with primary antibodies (1:1000 dilutions), including VP1 (GeneTex, China), p-MST1/2 (Affinity, USA), MST1/2 (Affinity, USA), p-LAST1/2 (Affinity, USA), LAST1/2 (Affinity, USA), p-YAP (Affinity, USA), YAP (Affinity, USA), TLR3 (Abclonal, China), TRIF, RIG-I (Abclonal, China), MDA5 (Abclonal, China), MAVS (Abcam, USA), TRAF3 (Abcam, USA), TBK1 (Abcam, USA), TLR7 (Abclonal, China), MyD88 (Abcam, USA), IRAK4 (Affinity, USA), IRAK1 (Affinity, USA), TRAF6 (Abcam, USA), TAK1 (Abcam, USA), NF-κB (CST, USA), IL-1β (CST, USA), IL-6 (CST, USA), IL-8 (CST, USA), TNF-α (CST, USA), IRF3 (Affinity, USA), IFN-β (Affinity, USA) and β-actin (Abbinke, China). After washed with TBST for three times, the membranes were probed with the respective horseradish peroxidase (HRP)-conjugated IgG secondary antibodies (1:10000 dilution; Abbinke, China). Finally, we examined protein signals through the enhanced chemiluminescence (ECL) system (Biosharp, China).

### Flow cytometry assay for the examination of inflammatory cytokines

Twelve inflammatory cytokines, namely, TNF-α, IL-12, IL-4, IL-17, IL-8, IFN-γ, IL-10, IL-1β, IL-6, IL-2, IFN-α and IL-5, are common cellular inflammatory factors in human immunity and play an important role in human immune regulation. In this study, we used a commercial Bio-Plex cytokine assay (RAISECARE, China) for testing according to the product specification. In brief, 25 µl sample, 25 µl trapped microspheres and 25 µl experimental buffer were mixed and incubated for 2 h by slightly shocking, followed by 25 µl of SA-PE antibody added and incubated for 30 min. After cleaning with 100 µl washing buffer and centrifuging for 5 min at 1500rmp/min, 300 µl washing buffer was supplemented and determined on the flow cytometer. Finally, the concentrations of inflammatory cytokines were calculated by a LEGENDplex v8.0 software.

### Immunofluorescence (IF) assay

For IF experiments, cells were seeded on poly-l-lysine coated coverslips. Following infection with CV-A10, cells were fixed with 4% paraformaldehyde and permeabilized with 0.1% Triton X-100 for 15 min at room temperature. Blocking is done with 2% bovine serum albumin (BSA; Biofroxx, China) for 1 h at room temperature prior to incubation with primary antibodies (1:100 dilution), including VP1, YAP, TBK1, IRAK1, NF-κB. Following thrice washes with PBS, the cells were further incubated with Alexa Fluor® 488-conjugated goat-anti-mouse IgG and Alexa Fluor® 594-conjugated goat-anti-rabbit IgG (1:300 dilution; CST, USA). After washing three times again, the coverslips were mounted on glass with anti-fade reagent which contained 4’,6-diamidino-2-phenylindole (DAPI; Beyotime, China). Fluorescent images were acquired on a confocal microscope (Leica, Germany).

### Statistics

All data are presented as the means ± SD at least three independent experiments. Statistical analysis was conducted using Graph Pad Prism software. When two groups were compared, the student’s t-test was applied. For three or more group comparisons, one or two-way analysis of variance (ANOVA) was performed, as appropriate, followed by Tukey’s or Bonferroni’s post hoc test, as appropriate.

## Results

### CV-A10 is susceptible to HMC3 cells

**Fig. 1:**
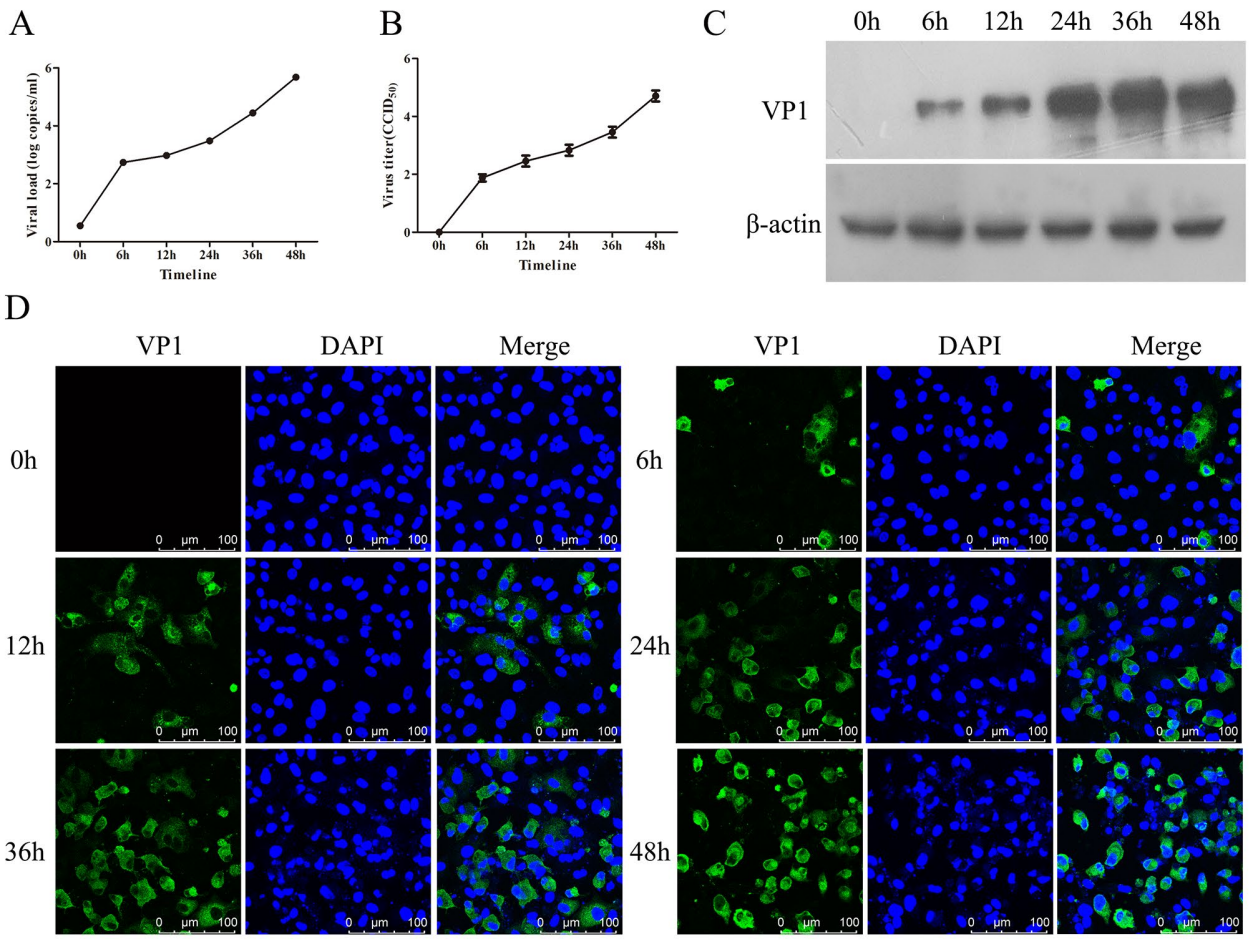
Proliferation kinetics of CV-A10 on HMC3 cells. **(A)** Viral load was examined by qRT-PCR. **(B)** Virus titer was calculated with the Reed-Muench method via TCID_50_ analysis. **(C)** VP1 protein expression level was measured by WB. **(D)** VP1 representing the presence of the virus in infected cells was monitored using IF staining

Nervous system damage has always been the cause of enterovirus infection [23]. As seen in Fig. 1, CV-A10 was gradually proliferated with time tested by viral load, virus titer and VP1 expression. Moreover, IF staining also exhibited that the infectious rate was elevated over time (Fig. 1D). Thus, these results suggested that CV-A10 could rapidly replicate in HMC3 cells, which provided a good model for in vitro cell study of microglia cell changes induced by CV-A10 infection.

### CV-A10 infection triggers inflammatory response in HMC3 cells

**Fig. 2:**
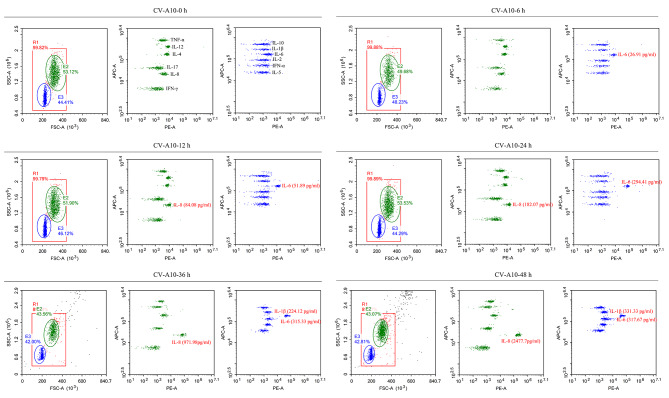
CV-A10 infection induces the release of inflammatory cytokines

A large number of studies have reported that the central nervous system lesions caused by enteroviruses infection not only lie in its replication, but also in the inflammatory changes of the nervous system caused by its replication [24]. So, we speculated that the inflammatory response induced by CV-A10 infection may also be one of the key reasons for triggering neuroinflammation. Our data revealed that IL-6 was the first to be released in CV-A10-infected HMC3 cells at 6 h after infection, then IL-8 was also found to begin to be released at 12 h after infection (Fig. 2). Moreover, IL-1β was detected to be up-regulated in the later stage of CV-A10 infection. Hence, these data indicated that inflammatory response might be activated in CV-A10-infected HMC3 cells.

### The formation of inflammatory response induced by CV-A10 is mainly dependent on the activation of innate immune pathways

**Fig. 3:**
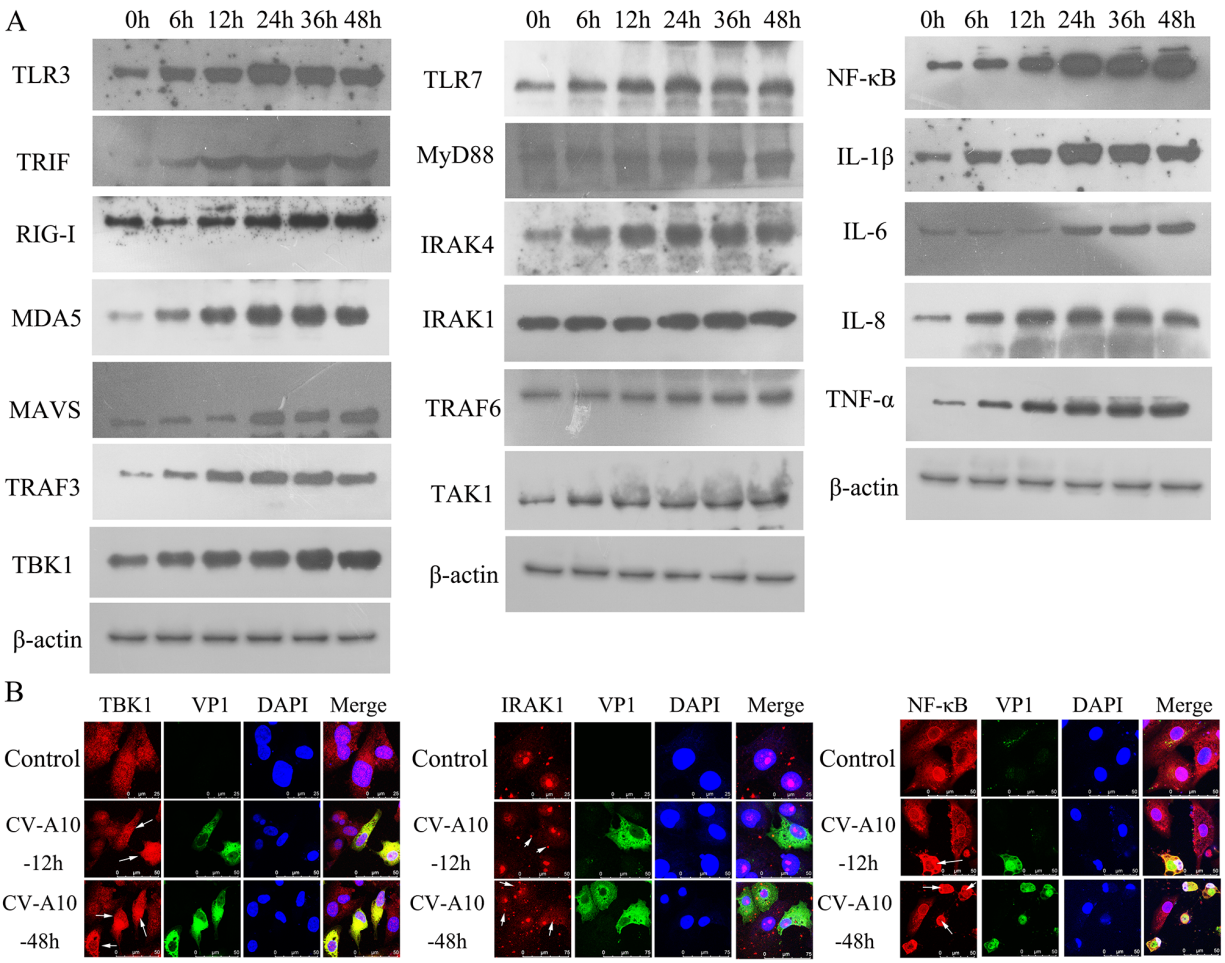
CV-A10 infection activates PRRs-mediated pathways. **(A)** WB analysis showed the expressions of innate immune pathway and inflammatory cytokines during CV-A10 infection. **(B)** IF images of HMC3 cells treated with CV-A10 at 12 h and 48 h time points illustrated TBK1, IRAK1 and NF-κB

NF-κB is a critical regulator of the immediate early pathogen response, playing an essential role for inflammatory response [25]. During viral infection, host innate immune system senses viral products, such as viral nucleic acids and viral proteins, to activate NF-κB signaling pathways [26]. In this study, we examined three innate immune pathways which can recognize viral RNA, namely TLR3-TRIF-TRAF3-TBK1-NF-κB axis, RIG-I/MDA5-MAVS-TRAF3-TBK1-NF-κB axis and TLR7-MyD88-IRAK1/IRAK4-TRAF6-TAK1-NF-κB axis. It was disclosed that the above proteins were time-dependently enhanced (Fig. 3A). Moreover, downstream proinflammatory cytokines mediated by NF-κB signaling pathway, such as IL-1β, IL-6, IL-8 and TNF-α, were also strengthened (Fig. 3A). Additionally, IF staining further observed that TBK1, IRAK1 and NF-κB were markedly elevated in CV-A10-infected cells (Fig. 3B). Thence, these data pointed out that CV-A10 might be recognized by pattern recognition receptors, such as TLR3, RIG-I, MDA5 and TLR7, on the host cells to stimulate subsequent inflammatory pathways.

### Hippo signaling pathway is dysregulated in CV-A10-infected cells

**Fig. 4:**
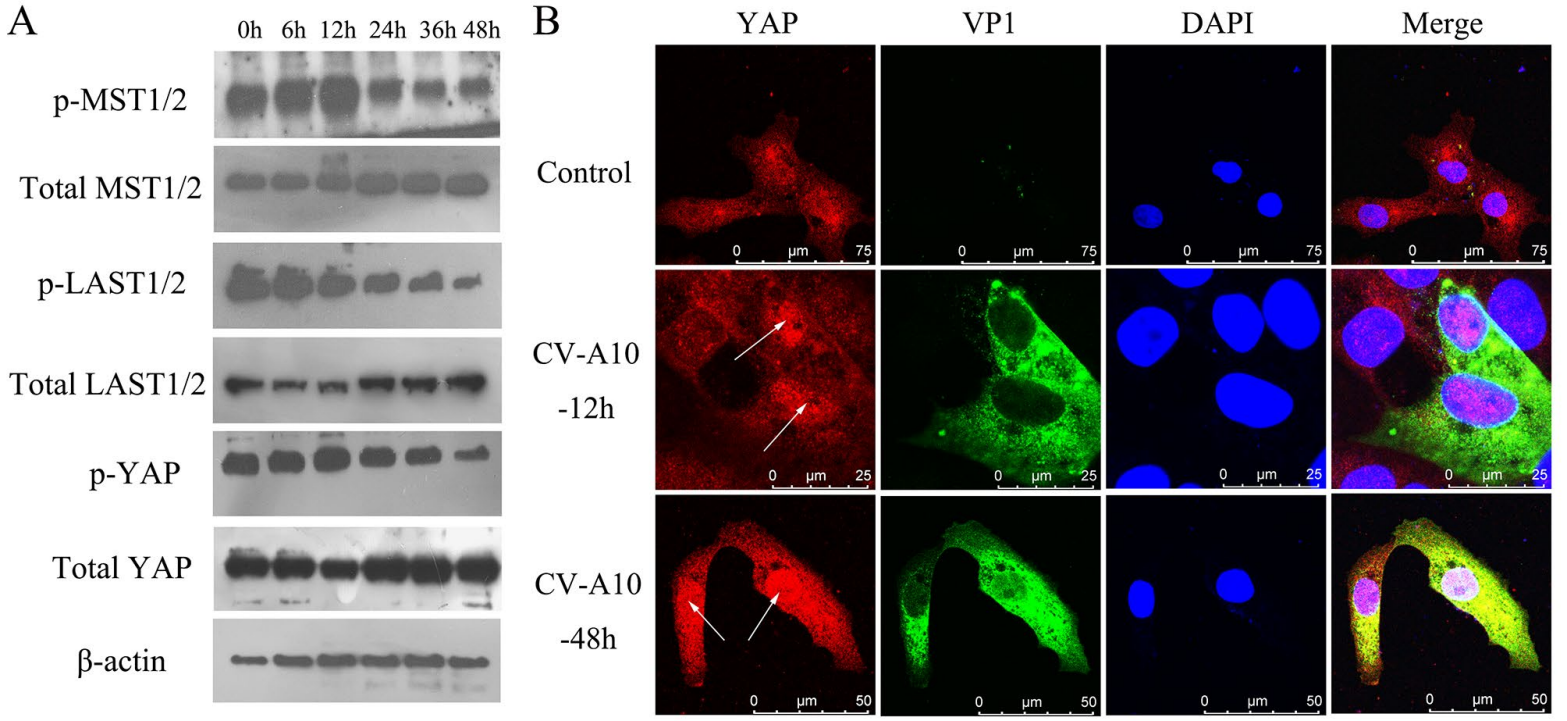
CV-A10 infection modulates Hippo signaling pathway. **(A)** WB analysis indicated the levels of Hippo signaling pathway in course of CV-A10 infection. **(B)** Representative images of two-color staining for YAP and VP1 in CV-A10-infected cells

The Hippo signaling pathway has long been considered as a tumor suppressor pathway [27], but recent studies have found that it also plays an important role in virus-induced diseases [19]. In this study, we analyzed these related molecules on the Hippo signaling pathway. WB results showed that p-MST1/2, p-LAST1/2 and p-YAP expressions were gradually down-regulated, while MST1/2, LAST1/2 and YAP expressions were correspondingly up-regulated slightly (Fig. 4A). Furthermore, translocation and accumulation of YAP in nucleus upon infection were observed in IF staining (Fig. 4B). Therefore, these findings implied that CV-A10 infection might inhibit Hippo pathway activation.

### MST1/2 is involved in the regulation of inflammatory response caused by CV-A10 infection

**Fig. 5:**
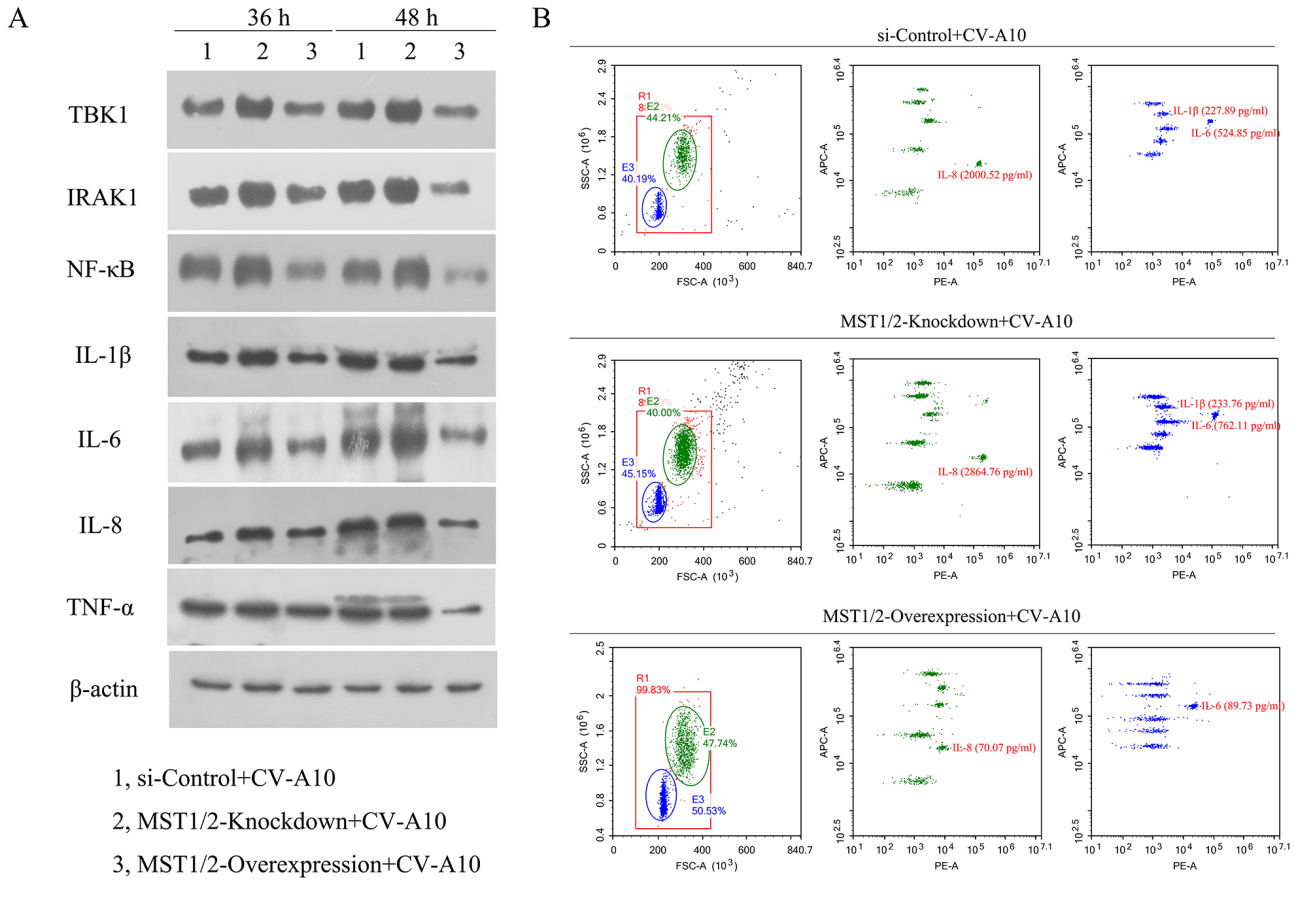
The effects of MST1/2 on inflammatory-related molecules. **(A)** MST1/2 acts on TBK1 and IRAK1 and influences the expression of inflammatory factors downstream. **(B)** MST1/2 inhibited the expressions of inflammatory cytokines after CV-A10 infection

Accumulating evidence has demonstrated that there is a crosstalk relationship between Hippo-YAP pathway and NF-κB signaling [13]. MST1/2 inhibits NF-κB activation via blocking TBK1 activation or promoting IRAK1 degradation [14]. Accordingly, we tranfected MST1/2-knockdown and MST1/2-overexpression vectors into cells. The transfection efficiency was also demonstrated to be significant (Fig. S1). Then, our data displayed that MST1/2-knockdown resulted in increasing levels of TBK1, IRAK1, NF-κB, IL-1β, IL-6 and TNF-α during CV-A10 infection, while MST1/2-overexpression leaded to decreasing levels of these proteins during CV-A10 infection (Fig. 5A). Meanwhile, flow cytometry also uncovered that MST1/2-knockdown aggravated the release of IL-6 and IL-8, but MST1/2-overexpression alleviated the release of IL-1β, IL-6 and IL-8 (Fig. 5B). Collectively, these results manifested that MST1/2 might regulate NF-κB-mediated inflammatory responses by acting on TBK1 and IRAK1.

### MST1/2 promotes CV-A10 replication in HMC3 cells

**Fig. 6:**
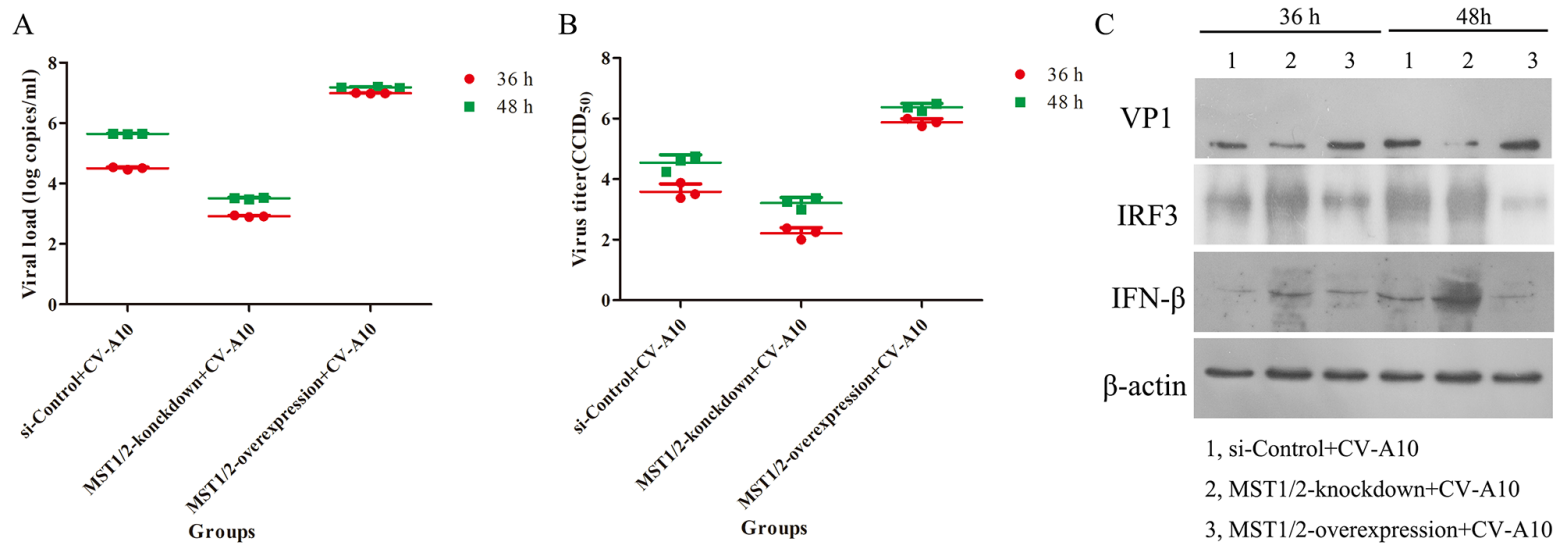
The influences of MST1/2 in virus production. **(A)** qRT-PCR was performed to evaluate the changes of viral load. **(B)** TCID_50_ assay was conducted to assess the changes of virus titer. **(C)** VP1, IRF3 and IFN-β in HMC3 cells after different treatments analyzed by WB

To further excavate the role of MST1/2 in CV-A10 replication, proliferation kinetics of CV-A10 was monitored. It was presented that MST1/2-knockdown dramatically resisted the viral load, virus titer and VP1 expression in CV-A10-infected cells, whereas MST1/2-overexpression sharply improved the above indicators in CV-A10-infected cells (Fig. 6). In consideration of the inhibitory role of MST1/2 on transcription factor IRF3, we guessed the overexpression of MST1/2 inhibited the expression of IRF3, which in turn further inhibited interferon production and ultimately promoted the replication of CV-A10. Thereafter, we tested the expressions of IRF3 and IFN-β. The results revealed that MST1/2-knockdown accelerated IRF3 and IFN-β expressions, but MST1/2-overexpression suppressed IRF3 and IFN-β expressions (Fig. 6C). Above all, these data hinted that MST1/2 improved viral replication of CV-A10, possibly by inhibiting IRF3.

## Discussion

Previous epidemiological data revealed that EV-A71 and CV-A16 are the most prevalent causal viruses for global HFMD outbreaks [3]. However, currently, incidences of CV-A10 infections have been an alarming trend in mainland China [8]. Moreover, CV-A10 has also been found to cause more serious inflammatory damage in the CNS [2, 9]. Under normal conditions, the CNS is an immune-privileged site because of a highly selective blood-brain barrier (BBB), but upon stimulation by neurotropic viruses, BBB may be destroyed, and cells in the CNS may also release some inflammatory cytokines that aggravate CNS lesions [28]. It has been found that microglia are the target of several neurotropic viruses [29] and microglia activation is the main driver of neuroinflammation in course of neurotropic viruses’ infection [30]. In this work, we put our attention on the microglia and chose HMC3 cells for experiments. On the one hand, we could observe the changes of CV-A10 after infection of major immune cells in the CNS; on the other hand, we could also speculate the potential pathological mechanism of CV-A10-induced neuroinflammation by the changes of CV-A10 after infection of microglia. And our results showed that CV-A10 could infect HMC3 cells and induce the release of inflammatory cytokines, especially IL-6, IL-8 and IL-1β. In fact, the moderate inflammatory response triggered by viral infection can play a role in eliminating the virus, but the persistent excessive inflammatory response is the key to causing tissue damage [31]. As illustrated with our results, the levels of IL-6, IL-8 and IL-1β gradually increased with the duration of CV-A10 infection. IL-6 is essential for the stimulation of immune cell activation and further production of inflammatory cytokines [32], IL-8 is believed to be involved in the pathophysiology of neurodegenerative illnesses [33, 34], while IL-1β causes neuronal damage and death through activation of the inflammasome and promotes BBB breakdown [35, 36]; thereby the changes of these cytokines in HMC3 cells might be implicated in the initiation and progression of neuroinflammation during CV-A10 infection. However, the regulation of inflammatory cytokines expression during viral infection is primarily dependent on the activation of pattern recognition receptors (PRRs) related pathways [37]. PRRs contain RIG-like receptors (RLRs), NOD-like receptors (NLRs), C-type lectin receptors, Toll-like receptors (TLRs) and DNA sensors, which can recognize a broad range of microbes, microbial products, and host derived damage-associated molecular patterns (DAMPs) [38]. Here, we only focus on the PRRs’ recognition of viruses. Previous studies have clearly reported that PRRs recognizes viruses mainly by identifying some viral proteins or viral nucleic acids [39]. In the PRRs that identify viral nucleic acids, it has been found TLR3 detects double stranded RNA (dsRNA), TLR7 detects single stranded RNA (ssRNA), while TLR9 detects hypomethylated CpG DNA [40]. Additionally, MDA5 detects long dsRNA, RIG-I detects short dsRNA, and cytosolic DNA sensors including absent in melanoma 2 and cyclic GMP-AMP synthase detect dsDNA [38, 40]. Thus, according to the above research, CV-A10, as a single stranded positive sense RNA [7], might be detected by some PRRs, such as TLR3, TLR7, RIG-I, MDA5. Then, these PRRs and their downstream signaling were examined. It was discovered that the expressions of receptor molecules, adaptor molecules, transcription molecules and downstream inflammatory cytokines in PRRs-mediated pathways showed an up-regulated trend. Meanwhile, key intermediate molecules mediating the production of inflammatory cytokines, such as TBK1, IRAK1 and NF-κB, also showed increasing or translocations into nucleus by IF staining. Hence, these findings suggested that CV-A10 infection of microglia might trigger the activation of PRRs-modulated inflammatory pathways. In fact, many studies on subsequent neuroinflammatory damage induced by some viruses’ infection of microglia also abound [30]. For instance, microglia are the main CNS cellular target for Human immunodeficiency virus 1 (HIV-1) infection and its activation contributes to neurotoxicity associated neurocognitive disorder [41]. Herpes simplex virus (HSV)-1 infection of microglia induces microglia to produce proinflammatory signals that promote neuronal loss without amplifying virus [42]. Therefore, to some extent, our results indicated that the inflammatory response activated by CV-A10 infection of microglia might be a key point in the neuroinflammatory injury induced by CV-A10 infection.

The Hippo signaling pathway, first described in Drosophila, is a tumor suppressor pathway which has been linked to various cancers [27]. Recent reports have also demonstrated that Hippo signaling pathway is involved in the regulation of inflammatory and immune responses against pathogens [14, 19]. Herein, we sought to investigate the regulatory role of this pathway in host’s inflammatory and immune responses during CV-A10 infection. When activated, Hippo signaling initiates a series of phosphorylation events via MST and LATS kinases, leading to the phosphorylation of YAP/TAZ which is sequestered in the cytoplasm by 14-3-3 proteins and further degraded; Vice versa, inactivation of Hippo signaling results in dephosphorylation of YAP/TAZ which then enters into nucleus for initiating transcription of its target genes [10, 11]. In this study, it was seen that phosphorylation of MST1/2, LAST1/2 and YAP were reduced, and YAP molecule was also translocated into the nucleus with CV-A10 infection time. Hence, these data indicated that CV-A10 infection suppressed the Hippo signaling. Previous study has reported that core kinases MST1/2 of Hippo signaling are extensively involved in the regulatory function in innate immune signaling. For example, MST1 inhibits the activation of TBK1, attenuating the subsequent NF-κB-mediated inflammatory gene expression [12]; MST1 can associate with IRAK1 to induce its phosphorylation and degradation, limiting the production of pro-inflammatory cytokines [13, 16]. Our study further provided evidences that knockdown of MST1/2 could reduce elevate TBK1 and IRAK1 expressions during CV-A10 infection, as well as their downstream inflammatory molecules, while overexpression of MST1/2 could reduce TBK1 and IRAK1 expressions, as well as their downstream inflammatory molecules during CV-A10 infection. Thus, these findings implied that MST1/2 might be a negative regulator of inflammatory response via repressing TBK1 and IRAK1. Nevertheless, downstream of TBK1 and IRAK1 are not only inflammatory pathways, but also antiviral pathways. The recognition of pathogens triggers the recruitment of adaptors to PRRs and initiates a series of protein kinases, including TBK1 and IRAK1, which further phosphorylates and activates downstream IRF3 to induce the synthesis and release of IFNs [37]. IFNs are important for host defence against viruses at an early stage [43, 44]. To excavate the effect of MST1/2 in virus production, viral load and virus titer were determined. It was obviously observed that depletion of MST1/2 downregulated virus production, but exogenously expression of MST1/2 upregulated virus production. Additionally, it was also displayed that depletion of MST1/2 restrained VP1 level and promoted IRF3 and IFN-β levels, whereas exogenously expression of MST1/2 reversed the above changes. Thence, combined with our above results, we can speculate that after CV-A10 infection of microglia, the cells would immediately initiate inflammatory and antiviral responses to resist the invasion of CV-A10 by inhibiting the activation of Hippo signaling pathway, but the overactivated inflammatory response of microglia caused by CV-A10 infection might became the basis of neuroinflammatory lesions with the prolonged duration of CV-A10 infection.

## Conclusion

Our study confirmed that CV-A10 infection induced the inflammatory and immune response via activating PRRs-mediated pathway and restricting Hippo signaling pathway in microglia, which may be the cause of neuroinflammatory lesions caused by CV-A10 infection of microglia. Meanwhile, based on the intricate interaction between innate immunity and Hippo signaling pathway, MST1/2 were demonstrated to involve in the regulation of the release of inflammatory cytokines and IFN-β via acting on TBK1 and IRAK1. Moreover, it was also verified that the effect of MST1/2 on IFN-β further had a certain impact on virus production, that is, the activation of MST1/2 enhanced CV-A10 infection. Taken together, this work further strengthens our mechanistic understanding of how CV-A10 intervenes in immune signaling by regulating Hippo signaling pathway to induce neuroinflammation and targeting of Hippo signaling pathway could be a new avenue in the management of CV-A10 infection. In this study, we investigated the effects of CV-A10 infection on microglia only at the cellular level, and these findings need to be further verified by animal models and preclinical or clinical studies in this regard, which would provide a new idea for the study of the pathogenesis of CV-A10 and a new target for the development of diagnosis and treatment measures of CV-A10.

### Electronic supplementary material

Below is the link to the electronic supplementary material.

Supplementary Material 1

Supplementary Material 2. Fig. S1 WB confirmation of MST1/2 expression in HMC3 cells under MST1/2-knockdown or MST1/2-overexpression treatments

## Data Availability

No datasets were generated or analysed during the current study.

## Abbreviations

CV-A10: Coxsackievirus-A10
HFMD: Hand, foot and mouth disease
CNS: central nervous system
PRRs: pattern recognition receptors
EV-A71: enterovirus 71
CV-A16: coxsackievirus A16
CV-A6: coxsackievirus-A6
MST1/2: Ste20-like kinases 1/2
LATS1/2: large tumor suppressor 1/2
YAP: Yes-associated protein
TAZ: transcriptional coactivator with PDZ-binding motif
TBK1: TANK-binding kinase 1
IRAK1: IL-1R-associated kinase 1
SARS-CoV-2: severe acute respiratory syndrome coronavirus type 2
NS4B: nonstructural protein 4B
HCV: hepatitis C virus
ZIKV: Zika virus
HSV-1: herpes simplex virus-1
HIV-1: human immunodeficiency virus 1
RLRs: RIG-like receptors
NLRs: NOD-like receptors
TLRs: Toll-like receptors
DAMPs: damage-associated molecular patterns
DMEM: Dulbecco’s Modified Eagles Medium
FBS: fetal bovine serum
MOI: multiplicity of infection
qRT-PCR: Quantitative real-time polymerase chain reaction
TCID_50_: 50% tissue culture infectious dose
PBS: phosphate-buffered saline
SDS-PAGE: sodium dodecyl sulfate polyacrylamide gel electrophoresis
PVDF: polyvinylidene difluoride
HRP: horseradish peroxidase
IF: Immunofluorescence
BSA: bovine serum albumin
BBB: blood-brain barrier
