# Supplementary figures and images for: MST1/2 exerts a pivotal role in inducing neuroinflammation and Coxsackievirus-A10 replication by interacting with innate immunity
Source: Virol J. 2024 Apr 19;21:89. doi: 10.1186/s12985-024-02355-5 (PMC11031903; doi:10.1186/s12985-024-02355-5)

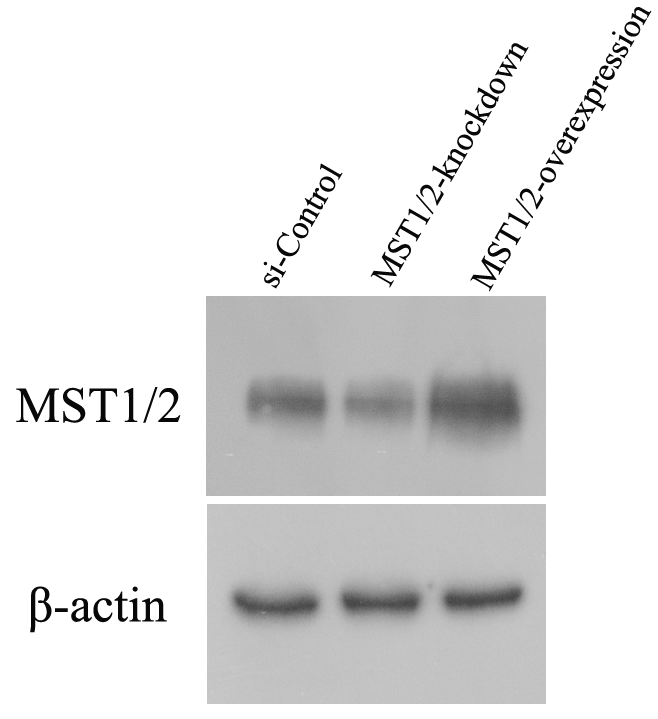

Supplement: Supplementary file 2 — Supplementary Material 2. Fig. S1 WB confirmation of MST1/2 expression in HMC3 cells under MST1/2-knockdown or MST1/2-overexpression treatments [file 12985_2024_2355_MOESM2_ESM.tif]
